# Sarcopenia in Inflammatory Bowel Disease: Prevalence, Mechanisms, Detection, Adverse Clinical Impact and Targetable Care Gaps—A Narrative Review Supported by a Structured Literature Search

**DOI:** 10.3390/life16091451

**Published:** 2026-08-31

**Authors:** Alexandra-Ioana Vasilachi-Lulache, Petruta Violeta Filip, Cosmin Alexandru Ciora, Eugen-Florin Georgescu, Laura Sorina Diaconu, Anca Roxana Băleanu, Corina Silvia Pop

**Affiliations:** 1 Faculty of Medicine, “Carol Davila” University of Medicine and Pharmacy, 050474 Bucharest, Romania; alexandra-ioana.lulache@drd.umfcd.ro (A.-I.V.-L.); sorina.diaconu@umfcd.ro (L.S.D.); cora.pop@umfcd.ro (C.S.P.); 2 Departments of Internal Medicine 3 and Gastroenterology, Bucharest University Emergency Hospital, 050098 Bucharest, Romania; 3 Gastroenterology & Hepatology Department, Fundeni Clinical Institute, 022328 Bucharest, Romania; cioracsz@yahoo.com; 4 Department of Surgery, University of Medicine and Pharmacy Craiova, 200349 Craiova, Romania; eugen.georgescu@umfcv.ro; 5 Department of Anesthesiology and Intensive Care, University Emergency Hospital Bucharest, 050098 Bucharest, Romania; ancaroxanapascal@gmail.com

**Keywords:** sarcopenia, low muscle mass, myosteatosis, inflammatory bowel disease, Crohn’s disease, ulcerative colitis, gut–muscle axis, body composition, muscle ultrasound, computed tomography, magnetic resonance imaging, artificial intelligence, nutrition, prognosis

## Abstract

Sarcopenia is increasingly recognized as a systemic complication of inflammatory bowel disease (IBD)—more accurately described as an IBD-associated muscle disorder than as a classical extraintestinal manifestation—but it remains inconsistently defined and rarely integrated into routine care. Consensus frameworks require low muscle strength confirmed by low muscle quantity or quality, so studies reporting only computed tomography (CT)-derived muscle area describe low muscle mass rather than consensus-defined sarcopenia; myosteatosis, the fat infiltration of muscle, is a further and partly independent dimension of muscle quality. This narrative review, supported by a structured literature search that was re-run and extended during peer review, synthesized peer-reviewed human evidence published from 1 January 2010 to 5 July 2026 in adult patients. Overall, 152 records were identified through PubMed/MEDLINE and citation tracking; after 19 duplicate or overlapping records were removed, 133 records were screened, 66 full-text reports were assessed, and 54 sources were included: 39 empirical IBD studies, 5 IBD-specific systematic reviews or meta-analyses, 6 consensus or standardization documents and 4 mechanistic or narrative reviews. Sarcopenia in IBD is driven by chronic inflammation, malnutrition, dysbiosis, corticosteroid exposure, inactivity and impaired anabolic signaling. Prevalence is definition- and setting-dependent, from approximately 10% in stable outpatients assessed with functional criteria to more than 40–50% in CT-based or active-disease cohorts. Sarcopenia is consistently associated with—rather than proven to cause—hospitalization, abscess formation, postoperative complications, treatment escalation or failure and impaired function. Muscle ultrasound and automated, artificial intelligence-assisted analysis of opportunistic CT and magnetic resonance imaging (MRI) are emerging as practical routes to routine assessment. We propose a drivers–detection–prognosis–intervention framework, aligned with the sequential European Working Group on Sarcopenia in Older People 2 (EWGSOP2) and Asian Working Group for Sarcopenia (AWGS) 2019 algorithms, to support opportunistic imaging review, strength testing and integrated nutrition–exercise care; this framework is an expert proposal that requires prospective, multicenter validation.

## 1. Introduction

Inflammatory bowel disease (IBD), comprising Crohn’s disease (CD) and ulcerative colitis (UC), is a chronic, relapsing immune-mediated disorder with intestinal and systemic consequences. Beyond mucosal inflammation, IBD affects nutritional status, bone health, fatigue, physical activity, mental health and work productivity. Modern management has moved from symptom control alone toward objective targets such as endoscopic healing, normalization of inflammatory markers and prevention of bowel damage [1,2,3]. Skeletal muscle belongs to this systemic picture. It is affected by the same inflammatory, nutritional and pharmacological processes that drive intestinal disease, carries prognostic information about surgery, hospitalization and treatment response, and is nevertheless almost never measured in routine IBD care.

Sarcopenia was initially framed as an age-related loss of muscle mass, but contemporary definitions treat it as a generalized skeletal muscle disease that combines low muscle strength, reduced muscle quantity or quality and, when severe, impaired physical performance [4,5,6,7]. Terminology therefore matters: low muscle mass, myopenia, myosteatosis and consensus-defined sarcopenia are not synonyms, and low muscle quantity alone does not establish a diagnosis of sarcopenia, which additionally requires impaired muscle strength, with impaired physical performance used to grade severity [4,5,7]. This conceptual shift is particularly relevant to IBD because many affected patients are young or middle-aged rather than geriatric. In IBD, muscle loss may coexist with normal or high body mass index (BMI), be masked by visceral adiposity or edema and remain undetected until surgery, hospitalization, treatment failure or prolonged fatigue occurs [8,9,10,11,12,13,14,15,16].

The clinical challenge is not merely that patients with IBD may lose muscle but that this loss occurs at the intersection of inflammation, nutrition, microbial ecology, pharmacology and physical function, while routine gastroenterology workflows rarely measure muscle health. Cross-sectional imaging is frequently obtained in IBD for diagnosis, complications or preoperative planning, yet the body-composition information contained in these examinations is commonly ignored. Conversely, bedside tools such as handgrip dynamometry, gait speed, the SARC-F questionnaire (Strength, Assistance in walking, Rise from a chair, Climb stairs and Falls) and calf circumference are inexpensive but rarely embedded in IBD clinics.

Previous reviews have addressed the prevalence and selected outcomes of sarcopenia in IBD, and recent meta-analyses have strengthened the association between sarcopenia and adverse outcomes [8,13,14,15,16]. However, several gaps remain. First, definitions have evolved from the European Working Group on Sarcopenia in Older People (EWGSOP) and Asian Working Group for Sarcopenia (AWGS) criteria to the Global Leadership Initiative in Sarcopenia (GLIS) conceptual definition, but IBD-specific thresholds are still lacking [4,5,6,7]. Second, mechanistic work on the gut–muscle axis is often separated from clinical diagnostic studies. Third, imaging-based and function-based sarcopenia phenotypes are frequently pooled despite measuring different dimensions of muscle health. Fourth, the care gap—under-screening despite modifiability—is rarely made operational. Fifth, muscle ultrasound, a low-cost, portable, radiation-free and repeatable bedside technique with a low environmental footprint that has recently undergone prospective validation in IBD, remains marginal in IBD reviews despite being well suited to longitudinal monitoring [17,18,19]. Sixth, muscle quality (myosteatosis) and automated, artificial intelligence-assisted image analysis are seldom integrated into the same conceptual framework as muscle quantity and muscle strength [20,21].

The objective of this review is therefore to synthesize the definitions, mechanisms, diagnostic approaches, prevalence patterns, prognostic implications and management opportunities for sarcopenia in IBD. The review is narrative rather than meta-analytic, but it is supported by a structured literature search and a PRISMA-like selection flow to improve transparency. Its practical contribution is a drivers–detection–prognosis–intervention framework that can guide opportunistic screening and future research in IBD.

## 2. Materials and Methods

### 2.1. Review Design and Rationale for the Timeframe

This article is a narrative review supported by a structured literature search. It was not registered as a systematic review, and no meta-analysis was performed because the included literature is heterogeneous in design, population, IBD subtype, diagnostic definition, imaging method, cut-off selection and outcome reporting. The PRISMA-like flow diagram is used as a reporting aid for transparency, not as a claim that the review is an exhaustive registered systematic review [22,23].

The search covered 1 January 2010 to 5 July 2026. In response to peer review, the search was re-run and extended in July 2026 with explicit terms for muscle ultrasound, diagnostic accuracy, reproducibility and automated image analysis, and all newly eligible diagnostic and prospective studies were incorporated into the evidence map. A 15-year interval was chosen deliberately. The lower boundary captures the modern sarcopenia era initiated by the 2010 European consensus and includes the subsequent EWGSOP2, AWGS 2019 and GLIS conceptual developments [4,5,6,7]. The upper boundary incorporates recent evidence through 2026, including CT-derived sarcopenia and myosteatosis studies using machine learning prediction models. Older landmark method papers were used only when necessary to explain imaging or body-composition concepts.

### 2.2. Information Sources and Search Strategy

The primary database was PubMed/MEDLINE. Additional records were identified through backward and forward citation tracking from key systematic reviews, meta-analyses and consensus papers. The PubMed/MEDLINE strategy combined controlled vocabulary and free-text terms:

(“Sarcopenia”[Mesh] OR sarcopenia[tiab] OR “low muscle mass”[tiab] OR myopenia[tiab] OR myosteatosis[tiab] OR “skeletal muscle index”[tiab] OR “skeletal muscle density”[tiab] OR “muscle attenuation”[tiab] OR “intermuscular adipose”[tiab] OR “body composition”[tiab] OR “muscle wasting”[tiab] OR “muscle loss”[tiab] OR “psoas muscle”[tiab] OR “muscle thickness”[tiab] OR “handgrip strength”[tiab]) AND (“Inflammatory Bowel Diseases”[Mesh] OR “inflammatory bowel disease”[tiab] OR IBD[tiab] OR “Crohn Disease”[Mesh] OR “Crohn disease”[tiab] OR “Crohn’s disease”[tiab] OR “Colitis, Ulcerative”[Mesh] OR “ulcerative colitis”[tiab]) AND (“1 January 2010” [Date-Publication]: “5 July 2026” [Date-Publication]) AND English[lang] AND Humans[Mesh].

A supplementary strategy was run to capture diagnostic, reproducibility and methodological studies that the main string under-retrieved: (ultrasound[tiab] OR ultrasonography[tiab] OR sonograph*[tiab] OR “artificial intelligence”[tiab] OR “machine learning”[tiab] OR “deep learning”[tiab] OR segmentation[tiab] OR radiomics[tiab] OR reproducibility[tiab] OR reliability[tiab] OR “diagnostic accuracy”[tiab] OR agreement[tiab]) AND (sarcopenia[tiab] OR “muscle mass”[tiab] OR “body composition”[tiab] OR myosteatosis[tiab]) AND (“inflammatory bowel disease”[tiab] OR IBD[tiab] OR “Crohn disease”[tiab] OR “Crohn’s disease”[tiab] OR “ulcerative colitis”[tiab]). Search terms were intentionally broad because IBD studies use variable terminology: sarcopenia, myopenia, low skeletal muscle index, psoas muscle index, myosteatosis and body composition are not interchangeable but are often used to describe overlapping muscle phenotypes. Records were exported to a screening log, de-duplicated manually and assessed in two stages: title/abstract screening followed by full-text eligibility assessment. Reference lists of all newly retrieved diagnostic studies and of the relevant standardization documents were additionally screened.

### 2.3. Eligibility Criteria

Eligible sources included peer-reviewed, English-language human studies and reviews addressing sarcopenia, low muscle mass, myopenia, myosteatosis or muscle function in adult patients with IBD. Original empirical studies were eligible if they reported prevalence, diagnostic methods, mechanistic correlates, treatment response, hospitalization, surgery, postoperative outcomes, fatigue, quality of life or follow-up outcomes. Systematic reviews, meta-analyses, consensus definitions and methodological papers were included when they informed definitions, diagnostic standards or evidence interpretation. Exclusion criteria were non-IBD populations without extractable IBD data, non-human studies used as primary evidence, conference abstracts without sufficient data, editorials or letters without original results, duplicated cohorts superseded by more complete reports, non-English full texts and studies focused only on unrelated metabolic or nutritional outcomes. Studies conducted exclusively in children or adolescents were also excluded, and this exclusion was deliberate rather than incidental: in growing patients, muscle mass must be normalized for height, pubertal stage and growth velocity and is interpreted against age- and sex-specific percentiles, so the adult consensus thresholds used throughout this review do not apply, and pooling the two bodies of literature would reintroduce precisely the definitional confusion the review is trying to remove. Every empirical IBD study included in the structured evidence map is therefore an adult study. Pediatric evidence is referred to in the Discussion (Section 4.2) for contrast only, and those references sit outside the structured search.

### 2.4. Selection, Extraction and Synthesis

For each eligible empirical source, the following items were extracted when available: first author, year, country or setting, design, IBD subtype, sample size, age profile, disease activity or surgical context, muscle assessment method, sarcopenia definition or cut-off, prevalence, clinical outcomes, adjusted effect estimates and principal methodological limitations. For systematic reviews and meta-analyses, database coverage, the number of included studies, pooled or narrative outcomes and conclusions were extracted. To avoid conflating constructs, every empirical study was additionally classified according to the muscle dimension actually measured: low muscle mass (muscle quantity only, for example, CT- or MRI-derived skeletal muscle index or psoas muscle index, dual-energy X-ray absorptiometry (DXA) appendicular lean mass or bioelectrical impedance analysis (BIA)-estimated skeletal muscle mass); low muscle quality (myosteatosis, for example, muscle attenuation, skeletal muscle density, intermuscular adipose tissue or MRI signal-intensity ratio); or consensus-defined sarcopenia (low muscle strength confirmed by low muscle quantity or quality, with or without impaired physical performance, according to EWGSOP2 or AWGS 2019). Throughout this review, the term applied to each study reflects that classification rather than the term used by the original authors. Because of heterogeneity, the synthesis was narrative and organized around four domains: definitions, mechanisms, diagnostic assessment and clinical/prognostic impact. Findings from high-level syntheses were prioritized for overall conclusions, while individual studies were used to illustrate phenotype, context and unresolved questions. The search and selection log is summarized in Table 1.

**Figure 1:**
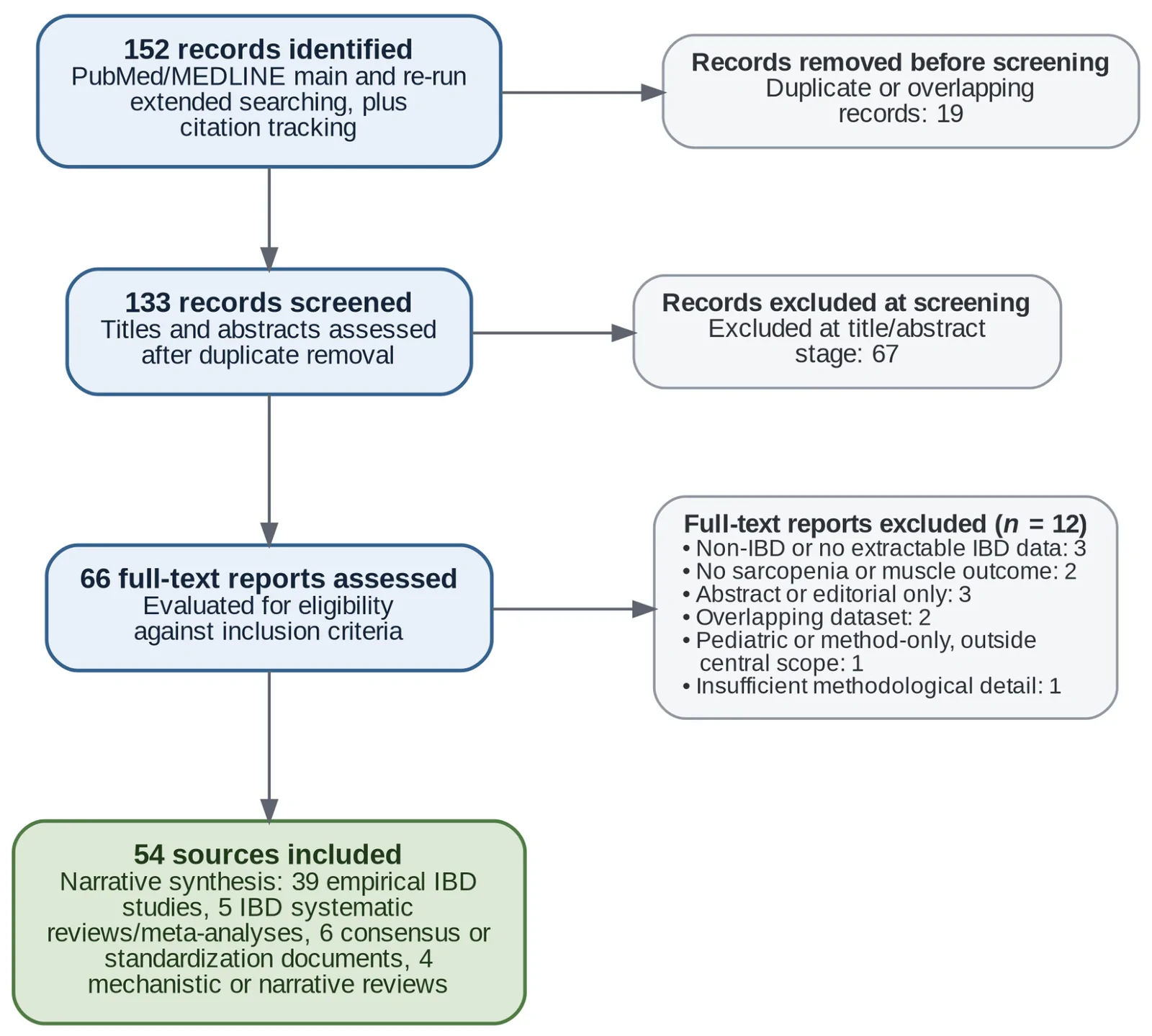
PRISMA-like flow diagram for record identification, screening and inclusion. The flow supports transparency for a narrative review; it does not convert the article into a registered systematic review.

The included empirical and synthetic evidence is summarized in Table 2, and the selection flow is summarized in Figure 1. The final included set was intentionally broader than a pooled meta-analysis dataset because the aim was integrative: consensus definitions, diagnostic methods, mechanistic reviews and clinical studies were all needed to answer the review question. To remove any numerical ambiguity between the text, Table 1 and Table 2, Figure 1 and the reference list, the counts reported in Table 1 and Figure 1 refer exclusively to records handled within the structured search. Of the 54 included sources, 44 are empirical IBD studies or IBD-specific systematic reviews and meta-analyses, and all 44 are tabulated individually in Table 2; the remaining 10 are consensus and standardization documents (EWGSOP2, GLIS, AWGS 2019, GLIM, the ESPEN clinical nutrition guideline and the SARCUS ultrasound framework) and mechanistic or narrative IBD reviews, none of which describes a cohort, a diagnostic definition or an outcome that could be tabulated, and all of which are cited in the text instead. The reference list additionally contains 27 background citations—general IBD epidemiology and treat-to-target sources, muscle biology and atrophy signaling papers, imaging and body-composition methodology, screening tool and nutrition or exercise sources, pediatric comparators and non-IBD technical or reporting papers—that were identified outside the structured search and are therefore deliberately not included in the PRISMA-like counts. The sum of the 54 included sources and these 27 background citations corresponds to the 81 numbered references (Figure 1).

## 3. Results and Narrative Synthesis

### 3.1. Evidence Map of Included Studies

Section 3 reports what the included literature contains and how it was measured; interpretation, reconciliation of divergent findings and clinical implications are deferred to Section 4, and the management discussion that previously closed Section 3 has been moved there for that reason. The included literature is dominated by observational cohorts, retrospective imaging studies and surgical or biologic therapy cohorts. Definitions vary substantially. Some studies used single-slice CT at the third lumbar vertebra (L3) to calculate the skeletal muscle index (SMI), some used the psoas muscle index (PMI), some used DXA, BIA or MRI, some quantified muscle quality as attenuation or a signal-intensity ratio, a small but growing group used muscle ultrasound, and more recent outpatient cohorts included strength and performance criteria. This methodological diversity is the main reason prevalence estimates differ widely and cannot be interpreted as interchangeable. Table 2 has therefore been reorganized by assessment modality and study objective rather than chronologically and reports for each source the design, the number of patients analyzed and the clinical setting, the muscle dimension actually measured with its definition and cut-off, the key result with adjusted estimates where available and the principal methodological limitation. It now covers all 44 empirical and synthetic IBD sources rather than a selection; the 10 remaining included sources are consensus, standardization and mechanistic documents that have no cohort to tabulate (Table 2).

### 3.2. Defining Sarcopenia in IBD: From Muscle Quantity to Muscle Disease

The definition of sarcopenia has moved from a muscle-mass construct toward a multidimensional muscle disease. EWGSOP2 places low muscle strength at the front of the diagnostic algorithm, uses low muscle quantity or quality to confirm the diagnosis and regards poor physical performance as a marker of severity [4]. AWGS 2019 similarly integrates strength, appendicular skeletal muscle mass and physical performance while adapting thresholds to Asian populations [7]. GLIS further broadens the field by proposing a global conceptual definition that includes muscle mass, muscle strength, muscle-specific strength and impaired physical performance as related but distinct components [5].

Four terms recur in this literature and must be kept distinct. Low muscle mass, or low muscle quantity, denotes a reduced amount of skeletal muscle, most often a low CT-derived skeletal muscle index at L3, a low psoas muscle index, low appendicular lean mass on DXA or a low estimated skeletal muscle mass on BIA. It is a single measurement, and on its own, it is not a diagnosis. Myopenia is used in parts of the IBD literature as a synonym for low muscle mass and carries exactly the same limitation. Myosteatosis denotes reduced muscle quality through ectopic fat accumulation within and between muscle fibers and muscle groups; on CT, it is quantified as low mean muscle attenuation or skeletal muscle density within the −29 to +150 Hounsfield unit window or as intermuscular adipose tissue and on MRI as an increased muscle-to-cerebrospinal fluid signal-intensity ratio. It can be present with entirely preserved muscle area, and no consensus numeric threshold for it currently exists [20,59]. Consensus-defined sarcopenia requires low muscle strength, confirmed by low muscle quantity or quality, with impaired physical performance defining severity [4,7]. Because the large majority of IBD studies report muscle quantity only, most of the prevalence and prognostic literature summarized below describes low muscle mass rather than consensus-defined sarcopenia; this distinction is applied consistently in the text and in Table 2, irrespective of the term used by the original authors.

This evolution matters in IBD because the literature has often used CT-derived low muscle mass alone as a proxy for sarcopenia. Such an approach is clinically attractive because CT scans are frequent in IBD, especially in CD and severe UC, but it conflates a single measurement with a multidimensional diagnosis and may overestimate or underestimate clinically meaningful muscle disease. Low L3 SMI identifies reduced muscle quantity; low muscle attenuation indicates myosteatosis and muscle quality; handgrip dynamometry identifies strength impairment; gait speed, chair stand or the Short Physical Performance Battery identify function. These domains overlap but should not be conflated.

In a young adult with active CD, low muscle mass may reflect inflammatory catabolism and malnutrition rather than aging. In an older UC patient, it may represent the interaction of IBD with primary sarcopenia, multimorbidity and inactivity. In a patient with overweight or obesity, sarcopenia may be hidden by high BMI, while visceral adiposity and ectopic muscle fat may magnify inflammation and treatment complexity. Consequently, borrowed geriatric, oncologic or population thresholds may not map perfectly onto IBD risk. The absence of IBD-specific cut-offs is one of the most consistent limitations across studies.

Both EWGSOP2 and AWGS 2019 are sequential rather than parallel algorithms, and this structure determines how the individual measures listed in Table 3 relate to one another. EWGSOP2 follows a find–assess–confirm–severity sequence: case-finding with SARC-F or clinical suspicion; assessment of muscle strength by handgrip dynamometry or the chair-stand test, which establishes probable sarcopenia and is by itself sufficient to trigger intervention; confirmation by low muscle quantity or quality on DXA, BIA, CT or MRI, which establishes the diagnosis; and severity grading by physical performance using gait speed, the Short Physical Performance Battery, Timed-Up-and-Go or the 400 m walk test [4]. AWGS 2019 preserves the same logic but adapts it to two settings: in community or primary-care settings, calf circumference, SARC-F or SARC-CalF trigger a strength or function test and permit a diagnosis of possible sarcopenia that can be managed without imaging, whereas in hospital or research settings, appendicular skeletal muscle mass is required for confirmation, and the combination of low strength and low physical performance defines severe sarcopenia [7]. Each individual measurement therefore has a defined position in the sequence rather than being one interchangeable option among many: SARC-F and calf circumference screen, handgrip strength and the chair-stand test establish probable or possible sarcopenia, imaging or BIA confirms it, and performance testing grades severity. For IBD, the practical consequence is twofold. Opportunistic CT contributes to the confirmation step but cannot complete the algorithm on its own, which is precisely why the CT-dominated IBD literature describes low muscle mass rather than sarcopenia. Conversely, the bedside strength test that constitutes the entry point of both algorithms is the element most often missing from IBD practice, even though it is the cheapest and quickest of all the measures involved.

### 3.3. The Gut–Muscle Axis: Mechanistic Drivers of Muscle Failure

Sarcopenia in IBD can be interpreted through a gut–muscle axis that integrates inflammation, nutrition, microbiota and physical activity. Active intestinal inflammation increases systemic cytokine exposure, including tumor necrosis factor-alpha and interleukin-6, which can activate catabolic pathways such as NF-kappaB and JAK/STAT3 signaling. These pathways increase ubiquitin-proteasome activity, including MuRF-1 and atrogin-1/MAFbx, and can reduce anabolic signaling through the IGF-1/PI3K/Akt/mTOR pathway [9,10,11,12,60,61,62].

Malnutrition amplifies these inflammatory signals. Reduced intake during flares, anorexia, abdominal pain, food avoidance, increased intestinal losses, malabsorption, low vitamin D status and micronutrient deficits can all reduce the substrate available for muscle protein synthesis [63,64]. Corticosteroids may further accelerate protein breakdown by suppressing IGF-1/PI3K/Akt/mTOR signaling and activating FoxO-dependent expression of atrogin-1 and MuRF-1 [65], while fatigue, pain, urgency, active disease and depression are the barriers most consistently reported by patients with IBD themselves as limiting physical activity [66,67]. Muscle then becomes both a target and a mediator of systemic illness: low muscle reduces functional reserve, worsens recovery from surgery and may alter the distribution and clearance of biologic therapies. Although not an IBD study, a 2026 cohort of adults with pulmonary tuberculosis and severe malnutrition found that a machine learning-assisted immune–inflammatory ratio based on routine leukocyte counts independently predicted in-hospital mortality, illustrating the prognostic importance of inflammatory dysregulation in a severely malnourished state [68].

**Figure 2:**
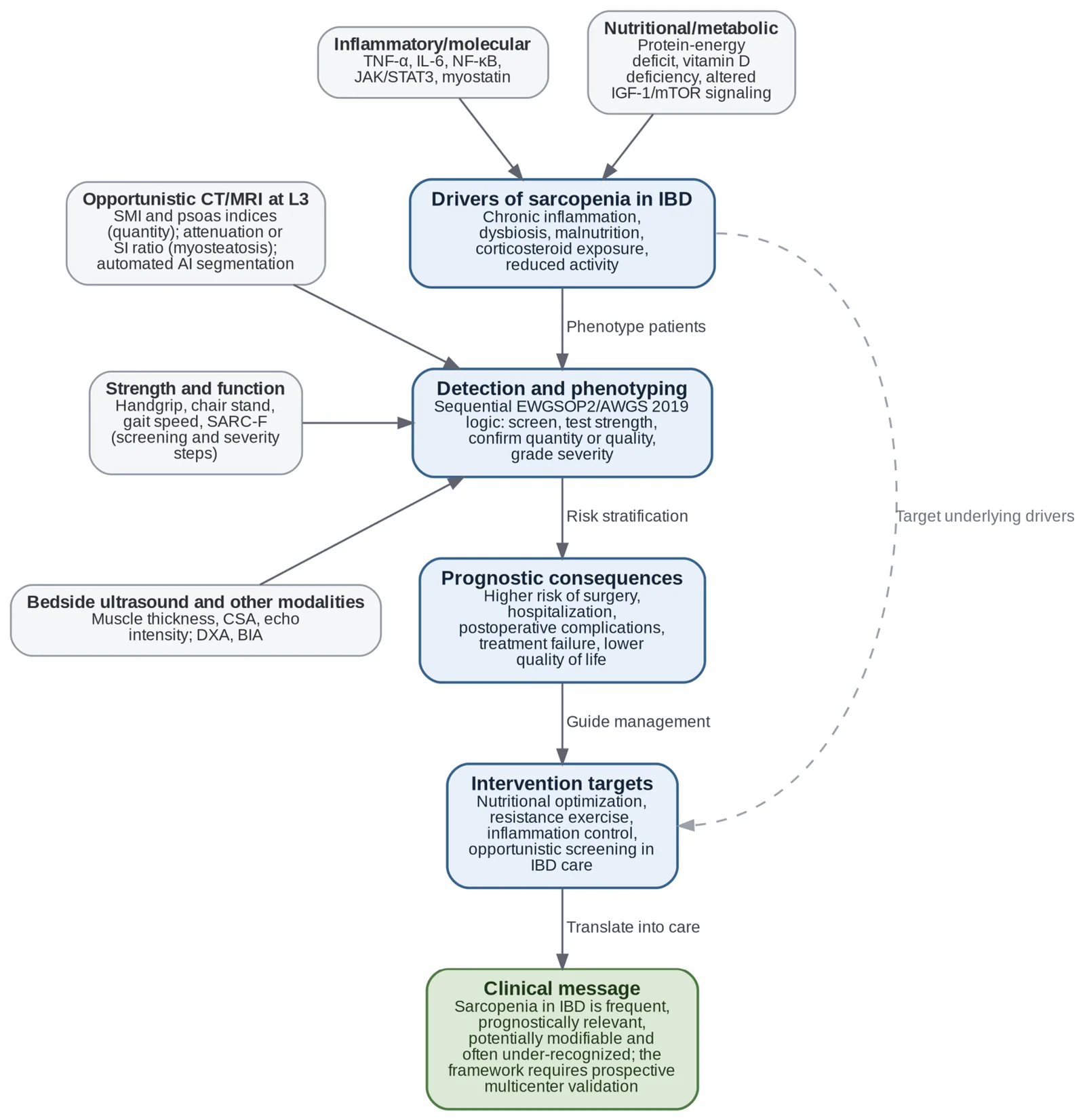
Drivers–detection–prognosis–intervention framework for sarcopenia in IBD. The model emphasizes under-recognition as the actionable care gap. Solid arrows indicate the proposed clinical sequence from drivers through detection and prognosis to intervention and care translation; the dashed arrow indicates feedback from intervention targets to the underlying drivers.

The gut microbiota provides another mechanistic bridge. Dysbiosis and impaired barrier function may reduce short-chain fatty acid production, increase exposure to microbial products such as lipopolysaccharide and sustain low-grade inflammation [10,11,12]. The gut–bone–muscle triad is also relevant because IBD is associated with osteopenia, osteoporosis, vitamin D deficiency and steroid exposure and because osteoporosis and sarcopenia share pathophysiology and cluster as osteosarcopenia, a combination that carries a higher risk of falls, fracture and mortality than either condition alone [64,69]. Ectopic lipid deposition links these processes to muscle quality: intermuscular and intramyocellular lipid accumulation impairs insulin signaling and mitochondrial function and is associated with reduced muscle-specific strength, which is why myosteatosis behaves as a partly independent dimension of muscle failure rather than as a late consequence of muscle loss [20]. Therefore, sarcopenic obesity and osteosarcopenia should be viewed not as secondary labels but as overlapping phenotypes within the same inflammatory–metabolic network; in IBD, sarcopenia has also been directly linked to metabolic comorbidity, including non-alcoholic fatty liver disease [40] (Figure 2).

### 3.4. Diagnostic Assessment: What Should Be Measured?

The most common IBD research method is cross-sectional imaging, particularly CT at L3. L3 skeletal muscle area correlates with whole-body skeletal muscle mass and permits calculation of SMI after normalization for height [70]. In IBD, this method is attractive because abdominal CT or CT enterography is often already available for clinical reasons. Opportunistic analysis can therefore extract additional prognostic information without new radiation exposure. The same acquisition also carries muscle quality information: mean muscle attenuation, conventionally measured within the −29 to +150 Hounsfield unit window, and intermuscular adipose tissue, delimited between −190 and −30 Hounsfield units, quantify myosteatosis, although the attenuation thresholds applied across studies remain inconsistent and limit comparability [20,59]. However, CT thresholds vary by sex, BMI, ethnicity, phase of contrast, segmentation software and reference population. Psoas-only indices are easier to obtain but may be less representative of total muscle than whole-slice L3 analysis.

MRI and MR enterography avoid radiation and are increasingly relevant in CD, especially for young patients and repeated imaging. MRI can quantify muscle area and, in some protocols, muscle fat infiltration, most often expressed as a muscle-to-cerebrospinal fluid signal-intensity ratio; these ratios are not numerically equivalent to CT attenuation and are not standardized across scanners or sequences [39,43]. DXA provides appendicular lean mass, is the confirmatory method recommended by both EWGSOP2 and AWGS 2019 and is the reference standard in most non-IBD sarcopenia research, but it gives little information on muscle quality and is not routinely performed in IBD unless bone density is being assessed [4,7,64]. BIA is inexpensive and accessible and is the alternative confirmatory method accepted by both consensus groups, but it estimates rather than measures muscle mass and is influenced by hydration, edema, recent food intake and disease activity, all of which are common problems in active IBD [4,7,42]. A structural asymmetry of the field deserves explicit emphasis here: in most other immune-mediated inflammatory diseases, muscle mass is assessed primarily by DXA or BIA, whereas the IBD evidence base is dominated by CT simply because abdominal cross-sectional imaging is generated as a by-product of routine care. This reflects data availability rather than a deliberate methodological choice, and it biases the IBD literature towards muscle quantity measured in sicker, imaged patients. Ultrasound, which is portable, radiation-free and repeatable, is considered separately in Section 3.4.1 because it is the modality with the largest gap between practical suitability and current use in IBD.

Muscle strength and performance are essential if sarcopenia is understood as a muscle disease rather than as low muscle area alone. Handgrip dynamometry is inexpensive, quick and compatible with EWGSOP2-style screening, and low grip strength is the entry criterion of both consensus algorithms [4,7]. Chair-stand testing, gait speed, the Short Physical Performance Battery and Timed-Up-and-Go add functional information and are used for severity grading [4,7]. SARC-F, a five-item self-report questionnaire covering strength, assistance in walking, rising from a chair, stair climbing and falls, has high specificity but low sensitivity in younger or less disabled populations [71]; SARC-CalF (SARC-F plus calf circumference) was developed specifically to correct that low sensitivity and improves case finding without an appreciable loss of specificity [72]. Because each of these measures occupies a defined position in the sequential consensus algorithms, Table 3 states not only what each modality measures but also whether it serves screening, confirmation or severity grading. The extent to which these measures are actually used in IBD can be read directly from Table 2. Of the 44 tabulated sources, 17 assessed muscle quantity alone on CT or MRI, 5 reported muscle quality, 3 used muscle ultrasound, 5 applied full consensus criteria combining strength with muscle mass, 5 related body composition to biologic therapy, 5 are systematic reviews or meta-analyses, and 4 are biomarker or automated-analysis studies. Within the 39 empirical studies, handgrip dynamometry was performed in only 8 and formal physical performance testing in 5, whereas CT-derived muscle quantity was measured in 25. That imbalance, rather than any disagreement about the definition of sarcopenia, is the practical reason why most IBD data describe low muscle mass. For IBD, a pragmatic pathway may therefore combine symptom and disease-risk triggers with handgrip strength and opportunistic imaging review rather than wait for a perfect universal test (Table 3).

#### 3.4.1. Muscle Ultrasound: An Underused Bedside Tool in Inflammatory Bowel Disease

Among the available modalities, muscle ultrasound shows the widest mismatch between its practical properties and its current use in IBD. It is inexpensive, portable, repeatable, radiation-free and has a low environmental footprint; it requires no patient transfer, no contrast and no ionizing radiation; and it can be performed at the bedside or in the outpatient clinic during the same visit as intestinal ultrasound, a technique already established in IBD practice and increasingly available in gastroenterology units. These properties suit two tasks that CT cannot perform. The first is serial monitoring of muscle status through flare, recovery, surgery and nutritional or exercise intervention, where repeated CT is neither justifiable nor safe. The second is the assessment of patients in whom cross-sectional imaging is not clinically indicated, which in IBD means most patients in sustained remission, precisely the group in which muscle deficits have been shown to persist unnoticed [46,50].

The reference standardization framework for muscle ultrasound is that of the SARCUS (Sarcopenia through Ultrasound) working group of the European Geriatric Medicine Society, which defined anatomical landmarks and measuring points for 39 muscles and specified five established parameters—muscle thickness, cross-sectional area, pennation angle, fascicle length and echo intensity—together with four emerging ones: muscle volume, stiffness assessed by elastography, contraction potential and microcirculation assessed by contrast-enhanced ultrasound [73]. The framework recommends taking the mean of three measurements for every item, standardizing patient position and reproducing it identically at follow-up, allowing a short rest period after repositioning and applying minimal transducer pressure to avoid compressing the muscle. Muscle thickness and cross-sectional area quantify muscle quantity, whereas echo intensity is the ultrasound correlate of myosteatosis, because fatty and fibrous infiltration increases muscle echogenicity. Important standardization gaps persist: echo intensity remains device- and setting-dependent with no accepted calibration phantom, the pennation angle shows considerable within-muscle variance, and the two published fascicle-length formulae have never been compared head to head [73]. Reference values are also incomplete and, where they exist, must be sex- and age-specific.

Reliability is the principal established strength of the technique. A systematic review of ultrasound muscle quantification in older adults reported intra- and inter-rater intraclass correlation coefficients spanning a wide range across studies, but with consistently high values of 0.72 to 1.00 for the muscles best suited to standardized protocols—vastus lateralis, rectus femoris, anterior upper arm and trunk—and validity correlations against reference methods of 0.92 to 0.999 [74]. A more recent scoping review restricted to studies applying contemporary consensus criteria identified only six studies and 24 validity tests in 1619 older adults; muscle thickness was the most frequently used parameter, EWGSOP2 and AWGS 2019 served as reference standards, and the highest discrimination was obtained for rectus femoris thickness, with an area under the curve of 0.92 (95% CI 0.89–0.94) [75]. That review also concluded that heterogeneity precluded meta-analysis and that validation in hospitalized patients remains lacking, two caveats that apply with equal force to IBD.

IBD-specific validation has now begun. In a prospective two-cohort study, 100 consecutive patients were used to establish feasibility and reliability, and a separate cohort of 53 patients with IBD to assess diagnostic accuracy against BIA-defined sarcopenia, with MRI as a second comparator [17]. The rectus femoris, rectus abdominis and biceps brachii were imaged with a high-frequency linear probe at defined anatomical landmarks, and an ultrasound muscle index was derived as the sum of the three thicknesses normalized to height squared. Inter- and intra-observer agreement was excellent, with single and mean intraclass correlation coefficients above 0.95 and a maximum of 0.97–0.98 for the rectus femoris cross-sectional area, and agreement between ultrasound and MRI for rectus abdominis thickness was likewise excellent (ICC 0.96). Discrimination for sarcopenia was good: rectus abdominis thickness reached an area under the curve of 0.85, biceps brachii and rectus femoris thickness 0.80 each, rectus femoris cross-sectional area 0.76 and composite ultrasound muscle index 0.81, with sensitivities of 80–100% at Youden-optimal cut-offs. All ultrasound parameters outperformed SARC-F (AUC 0.54) and the chair-stand test (AUC 0.76) in the same population, which positions ultrasound as a screening rather than a confirmatory test in this setting.

Two further IBD studies extend these observations in different directions. A prospective single-center study screened 353 patients with IBD using SARC-F and performed muscle ultrasound in the 57 patients identified as at risk; a diaphragm thickness index correlated with the psoas muscle thickness-to-height ratio (r = 0.36, *p* < 0.05), an association that persisted after adjustment (beta 0.018, 95% CI 0.005–0.030, *p* = 0.008) and was unaffected by age or sex, with inter- and intra-rater reliability again excellent (ICC > 0.95); the authors proposed the diaphragm as an accessible surrogate measurement site [18]. A hospital-based comparative study of 102 patients with IBD and 10 controls found that ultrasound-measured mid-thigh muscle thickness was significantly lower in patients than in controls and correlated with anthropometric mid-thigh circumference, BIA-derived skeletal muscle mass and handgrip strength, although that study reported neither diagnostic accuracy nor reliability statistics [19]. Taken together, the IBD-specific ultrasound evidence consists of three prospective single-center studies with a combined sample in the low hundreds, one of which is descriptive rather than diagnostic.

The current limitations are therefore explicit and should be stated rather than glossed over. Muscle ultrasound is operator-dependent, and although the reported reliability is excellent, it was generated by trained operators working to a protocol, so it cannot be assumed to transfer unchanged to unselected practice. No IBD-specific cut-offs exist: the published thresholds are either borrowed or derived in-sample against BIA reference standards that themselves rely on non-IBD and, in one instance, Asian-derived criteria applied to a European population [17]. The muscle quality parameters recommended by SARCUS—echo intensity, pennation angle, fascicle length, stiffness and microcirculation—have not been assessed in any IBD cohort, so ultrasound-based assessment of myosteatosis in IBD is at present entirely unexplored. Fluid shifts, peripheral edema and abdominal distension during active disease may affect thickness measurements in much the same way that they affect BIA, and this has not been formally quantified in IBD. Finally, and most importantly, no study has yet linked ultrasound-defined muscle status in IBD to hard clinical outcomes such as surgery, hospitalization, treatment failure or flare-free survival. The realistic near-term role of muscle ultrasound in IBD is therefore that of a repeatable screening and monitoring tool embedded within existing intestinal ultrasound practice, pending multicenter validation with outcome linkage, standardized acquisition and IBD-specific thresholds.

#### 3.4.2. Automated and Artificial Intelligence-Assisted Image Analysis

Manual or semi-automated segmentation is the single largest practical obstacle to routine opportunistic body-composition assessment: a trained reader is required, throughput is low, and additional inter-observer variability is introduced at the segmentation step, on top of the variability already contributed by the contrast phase, reconstruction kernel and threshold selection. Automated analysis removes this bottleneck. General-purpose deep learning segmenters trained on large multi-organ CT datasets now achieve Dice coefficients above 0.94 for anatomical structures including skeletal muscle [76], and open pipelines can identify the L3 vertebral level, extract the corresponding slice and segment muscle and adipose compartments without human input.

Evidence in gastrointestinal and specifically IBD populations is now emerging. A deep learning pipeline combining automated vertebral localization with nnU-Net muscle segmentation was trained on 550 CT scans and tested on 601 scans spanning acute pancreatitis, IBD, gallbladder cancer and biliary obstruction; it achieved Dice coefficients of 0.93–0.97, expert-rated excellent muscle segmentation in 90–93% of cases, and sarcopenia detection with a sensitivity of 0.94–0.97, a specificity of 0.84–0.97 and an area under the curve of up to 0.92 across heterogeneous protocols, contrast phases and radiation doses [21]. In Crohn’s disease, an automated CT-enterography segmentation algorithm was used to derive a skeletal muscle ratio expressing the balance between skeletal muscle and intermuscular adipose tissue in 157 patients; this ratio discriminated treatment escalation better than conventional sarcopenia metrics (AUC 0.82 in penetrating and 0.92 in non-penetrating disease) and remained an independent protective factor in Cox models (combined HR 0.64, 95% CI 0.49–0.82, *p* = 0.001) [45]. Radiomic analysis of the psoas muscle on CT has been used to predict infliximab response in 134 patients with Crohn’s disease, with a mean validation area under the curve of 0.85 across seven algorithms and 0.91 for the best-performing gradient-boosting model [58], and a machine learning model combining CT-derived skeletal muscle index and skeletal muscle density predicted treatment escalation in hospitalized patients with IBD, with a validation area under the curve of 0.763 [57].

The case for automation is strengthened by data from outside IBD that show what is discarded when body composition is not analyzed. In 9223 asymptomatic adults undergoing screening CT, fully automated muscle density—that is, muscle quality—predicted five-year mortality with an area under the curve of 0.721 (95% CI 0.683–0.759) and a hazard ratio of 3.58 (95% CI 3.02–4.23) for the highest-risk quartile, whereas body mass index was essentially non-discriminatory (AUC 0.499) [77]. This is precisely the information currently lost every time an abdominal CT performed for an IBD indication is reported without body-composition analysis. Several caveats nevertheless apply before clinical adoption. Most IBD studies are retrospective, single or two center and internally rather than externally validated; radiomic features in particular are sensitive to the scanner, reconstruction kernel and contrast phase; the sarcopenia labels used to train these models still depend on borrowed cut-offs, so an accurate model may faithfully reproduce an imprecise definition; and psoas-only regions of interest remain a weak surrogate for total lumbar muscle. Regulatory approval, integration into the radiology reporting workflow and prospective demonstration that automated flagging actually changes management are all outstanding. Even so, automated segmentation is the most plausible mechanism by which opportunistic muscle assessment could become routine in IBD, and it deserves to be evaluated as an implementation strategy—for example, as an automatically generated body-composition field appended to every abdominal CT or MR enterography report in patients with IBD—and not only as a prediction exercise.

### 3.5. Prevalence and Clinical Phenotypes

Prevalence is not a single stable number in IBD, and it should never be reported without the definition, modality and clinical setting that produced it. Stratified in this way, three bands emerge from the included literature. First, when only muscle quantity is measured on CT in hospitalized, active or preoperative cohorts, low muscle mass is reported in approximately 40–60% of patients [8,24,25,30,31,32,34,35,36,37,42,44]. Second, when consensus criteria combining strength and muscle mass are applied in mixed inpatient–outpatient or follow-up cohorts, sarcopenia is reported in approximately 20–50%, with the spread driven largely by whether AWGS 2019 or EWGSOP2 thresholds were used [28,34,35]. Third, when the same consensus criteria are applied to stable outpatients in remission, sarcopenia falls to approximately 10%, with probable sarcopenia in a further 18% and myopenia in 20% [50]. Against this background, the 2019 systematic review reported myopenia in 42% of patients and sarcopenia proper in 17% across five studies, and the 2023 systematic review found that available studies remained heterogeneous in population, criteria and outcomes [8,13]. Ultrasound-based estimates are still too few to define a band; the single IBD diagnostic accuracy cohort reported a 50% prevalence against BIA-based criteria in a hospital population [17].

Several clinical phenotypes can be distinguished. First, active inflammatory sarcopenia is seen in patients with high disease activity, hospitalization, acute severe UC or complicated CD [38,48]. Second, post-surgical or preoperative sarcopenia marks reduced physiological reserve and may indicate higher morbidity risk. Third, sarcopenic obesity combines low muscle with high fat mass or visceral adiposity; this phenotype is especially likely to be missed by BMI. Fourth, remission sarcopenia shows that muscle deficits can persist despite clinical symptom control. Fifth, myosteatosis may carry prognostic information even when muscle area is not severely reduced, suggesting that muscle quality is not merely a secondary detail [41,42,44,45].

The wide prevalence range should not be read as contradictory evidence that sarcopenia is either rare or universal; rather, it reflects the measurement of different constructs with different thresholds, using different modalities and across different clinical contexts. A hospitalized CT-based cohort of active IBD is not comparable to a remission outpatient cohort assessed by handgrip strength, performance tests and BIA. For publication and clinical use, studies should report the exact definition, cut-off, modality, imaging level, segmentation approach, disease activity and nutritional context and should state explicitly whether the reported figure refers to low muscle mass or to consensus-defined sarcopenia.

### 3.6. Prognostic Impact: What Outcomes Are Most Consistent?

The most consistent finding is that sarcopenia identifies a vulnerable IBD phenotype. Early evidence associated sarcopenia with surgery and postoperative complications [8,24,25,26,27,28,34,35,36,37,42,44]. Later meta-analytic evidence strengthened but also refined this signal. A 2024 meta-analysis of 17 studies and 2895 IBD patients found that sarcopenia was associated with treatment failure (OR 2.00, 95% CI 1.43–2.79) and with the need for surgery (OR 1.54, 95% CI 1.06–2.23), while subgroup analyses for pharmacologic plan change, corticosteroids and biologics alone were less consistent [15]. A 2025 CD-focused meta-analysis including 14 studies and 2334 patients found a higher hospitalization risk (OR 1.87, 95% CI 1.19–2.93) and abscess formation (OR 5.03, 95% CI 2.05–12.38) but no statistically significant pooled effect on surgery, loss of biological response, need for biologic therapy or surgical-site leak [16].

Muscle quality carries prognostic information that is partly independent of muscle quantity, and this is one reason why studies restricted to muscle area give inconsistent results. In Crohn’s disease, lower mean muscle attenuation was independently associated with a complicated stricturing or penetrating phenotype (OR 0.81 per unit increase in attenuation, *p* = 0.002), alongside a high visceral fat index (OR 26.1, *p* = 0.02) [41]. In operative Crohn’s disease, intermuscular adipose tissue was independently associated with postoperative morbidity (OR 1.08, 95% CI 1.01–1.16, *p* = 0.037) and with a higher comprehensive complications index, whereas adiposity alone was not, suggesting that it is fat within muscle rather than fat in general that marks risk [44]. On MR enterography, myosteatosis defined by a psoas-to-cerebrospinal fluid signal-intensity ratio was associated with anti-TNF initiation and low muscle mass with abscess and need for surgery (adjusted OR 5.34, 95% CI 1.02–28.03, an estimate whose width should temper its interpretation) [43]. The evidence is not uniformly positive: a retrospective cohort of 223 patients undergoing ileocecal resection found that neither MRI-defined myopenia nor MRI-defined myosteatosis independently predicted anastomotic leak, postoperative complications or disease recurrence [42]. That discrepancy is instructive rather than contradictory because CT attenuation thresholds and MRI signal-intensity ratios are not equivalent measurements, quartile-derived cut-offs differ between cohorts, and no consensus numeric definition of myosteatosis exists [20,59]. Muscle quality should therefore be reported alongside muscle area in future IBD studies, but it should not yet be treated as a validated risk criterion.

These results should be interpreted as prognostic associations, not as proof of causation, and the wording used throughout this review reflects that limitation. Sarcopenia may directly impair wound healing, immune function, mobility and recovery, and it may also be a marker of more severe inflammatory burden, long disease duration, malnutrition, steroid exposure or occult complications; both mechanisms are proposed in the IBD-specific reviews and are compatible with the meta-analytic pattern of strong associations with global outcomes and weaker, inconsistent associations with procedure-specific ones [9,12,15,16]. Both interpretations can be clinically useful: a marker that identifies high-risk patients may still guide screening, nutritional intervention, prehabilitation and treatment intensity, even before causality is established.

Treatment response is a particularly important but unsettled area. Low muscle mass may affect biologic pharmacokinetics, distribution volume and drug clearance, while systemic inflammation and albumin loss also influence drug exposure. Observational studies suggest associations between low muscle mass and anti-TNF failure or biologic loss of response, but meta-analytic subgroup findings remain mixed [15,51,52,53,54,55]. On present evidence, sarcopenia is best regarded as a risk marker when planning biologic therapy rather than as a standalone criterion for dose selection.

Quality of life, fatigue and functional outcomes are under-reported relative to surgery and hospitalization. This is a major gap because muscle disease affects daily functioning even when classic IBD endpoints improve. Fatigue, reduced mobility, low exercise tolerance and fear of eating or moving may perpetuate deconditioning. Future studies should integrate patient-reported outcomes and performance measures rather than rely only on imaging endpoints.

## 4. Discussion

### 4.1. Integrative Interpretation

This review supports three conclusions. First, sarcopenia in IBD is biologically plausible and clinically frequent, but its measured prevalence depends heavily on the definition, modality and clinical setting. Second, the prognostic signal is strongest for global adverse outcomes such as hospitalization, abscess, postoperative morbidity, treatment escalation and treatment failure, while some outcome-specific associations remain inconsistent. Third, the main translational gap is not a lack of available tools; it is the lack of standardized implementation. IBD clinicians already have access to risk factors, nutritional assessment, inflammatory biomarkers, strength testing, bedside ultrasound and often cross-sectional imaging, yet these data are rarely integrated into a muscle health pathway.

The drivers–detection–prognosis–intervention framework proposed here makes the care gap operational. Drivers include inflammation, malnutrition, dysbiosis, steroids, inactivity and aging. Detection requires separating muscle quantity, muscle quality, strength and performance. Prognosis requires recognizing that sarcopenia is not only a descriptive body-composition variable but also a risk marker for adverse outcomes. Intervention requires closing the loop through disease control, nutrition, resistance exercise, steroid stewardship, prehabilitation and follow-up measurement.

Because this framework is an expert proposal rather than a validated instrument, the route by which it could be tested should be stated explicitly. A realistic validation strategy has three stages. The first is descriptive and diagnostic: a prospective, multicenter cohort of consecutive patients with IBD, enrolled across remission, flare, preoperative and biologic-initiation settings, in which all four detection domains are measured concurrently—muscle quantity by opportunistic CT or MRI where already available, muscle quality by attenuation or signal-intensity ratio, muscle strength by handgrip dynamometry and physical function by gait speed or the chair-stand test—together with bedside muscle ultrasound, disease activity, nutritional status assessed against GLIM criteria, steroid exposure and patient-reported outcomes. A cohort of this design would allow for IBD-specific, outcome-anchored thresholds to be derived rather than borrowed and would quantify the agreement between domains that the current literature simply assumes. The second stage is prognostic: following the same cohort for at least 12 to 24 months would allow the framework’s detection output to be tested against hospitalization, surgery, treatment escalation, flare-free survival, fatigue and quality of life, with pre-specified adjustment for disease activity, albumin, steroid exposure, disease duration and prior surgery, so that muscle status can be distinguished from a general marker of disease severity; discrimination and calibration of a framework-derived risk score should be reported and then externally validated in an independent cohort. The third stage is implementation: a stepped-wedge or cluster-randomized trial in which IBD centers introduce the pathway—opportunistic imaging review with automated segmentation, a bedside strength test at defined triggers and structured referral to dietetics and physiotherapy when abnormal—with a clinical or patient-reported primary endpoint rather than a body-composition endpoint, as well as with feasibility endpoints such as the proportion of eligible patients screened, the consultation time added and the referral completion rate. Only the third stage can establish whether implementing the framework improves outcomes; the first two establish whether it measures what it claims to measure and identifies the patients it claims to identify. Until at least the first two stages are complete, the framework should be used to structure research and audit rather than to direct individual patient care.

### 4.2. Why the Literature Diverges

Divergence in the literature is expected. Studies differ by IBD subtype, activity, age, inpatient versus outpatient setting, surgical context, imaging availability and definition. CT-based cohorts often include sicker patients and detect low muscle quantity; functional cohorts may identify fewer but clinically more specific cases. CD is over-represented because CT and MR enterography are common, while UC evidence is expanding but remains less mature. Some studies use sarcopenia to mean low SMI only, whereas others require low strength with performance impairment, and the two systematic reviews that examined this question directly both concluded that the diagnostic criteria and cut-offs applied across IBD studies are too heterogeneous to pool [13,14]. This definitional problem explains why one study can report sarcopenia in about 10% of stable outpatients assessed with functional criteria while another reports rates above 40% in active or imaging-based cohorts [8,50].

Outcome divergence also reflects confounding. Sarcopenia may coexist with severe inflammation, low albumin, anemia, steroid exposure, strictures, penetrating disease or prior surgery, and not all studies adjust for these factors. Some outcome categories, such as hospitalization and abscess, may be more sensitive to systemic reserve and disease severity, while surgical leak or biologic response may depend on operative technique, drug pharmacokinetics, albumin, inflammatory burden and treatment timing; this is the most economical explanation for the pattern in the two most recent meta-analyses, in which hospitalization and abscess were significantly associated with sarcopenia but surgical-site leak and loss of biological response were not, and for the negative postoperative findings in the two cohorts that adjusted most thoroughly for operative and nutritional covariates [15,16,31,42]. Modality is a further and easily overlooked source of divergence: CT and MRI measure muscle area on a single lumbar slice, DXA measures appendicular lean mass, BIA estimates fat-free mass from impedance and is sensitive to hydration, and ultrasound measures regional thickness or cross-sectional area. These are correlated but not interchangeable quantities, each with its own cut-off set and its own failure modes in active disease. Therefore, a single pooled effect across all outcomes and all modalities may be less informative than phenotype- and modality-specific risk models.

Adult and pediatric evidence should be read separately rather than pooled, and the restriction of this review to adults is a deliberate boundary rather than an oversight. A distinct pediatric study exists and points in the same direction: in 101 children with IBD assessed by MR enterography, psoas muscle area indexed to body surface area was lower than in controls, and children in the lowest quartile had a higher risk of biologic therapy and disease exacerbation [78]; in 78 children with newly diagnosed Crohn’s disease, sarcopenia at diagnosis was present in 59% and independently predicted clinical relapse at 6 and 12 months [79]. The measurement problem is nevertheless different in kind. Muscle mass in a growing child must be normalized for height, pubertal stage and growth velocity and is interpreted against age- and sex-specific percentiles, so the adult cut-offs discussed throughout this review are not transferable; MRI rather than CT is the imaging modality of choice for radiation reasons; grip-strength reference values are age-dependent; and impaired linear growth is a competing outcome that has no adult equivalent. Reporting the two studies together would therefore reintroduce exactly the definitional heterogeneity that this review argues against. The practical implication is that the framework proposed here applies to adults and that a pediatric version would need its own thresholds, its own outcome set and its own validation.

### 4.3. Management and Therapeutic Targets

No IBD-specific sarcopenia treatment pathway has been validated in large, randomized trials. Nevertheless, the intervention logic is clear. Management should combine control of intestinal inflammation, adequate protein-energy intake, correction of micronutrient deficiency, resistance exercise, reduction of unnecessary corticosteroid exposure and early prehabilitation for patients approaching surgery. In active disease, nutrition and exercise prescriptions must be individualized to symptoms, strictures, anemia, fatigue, bone health and steroid exposure [63,64].

Protein targets discussed for sarcopenia are higher than standard adult minimums: the PROT-AGE position paper recommends at least 1.0–1.2 g/kg/day in healthy older adults and 1.2–1.5 g/kg/day during acute or chronic illness, combined with resistance exercise, and the ESPEN guideline applies a comparable logic to IBD [64,80]. In IBD, however, protein adequacy cannot be separated from disease phenotype: stricturing disease, short bowel, postoperative state, active diarrhea and food avoidance require dietetic input. Vitamin D and bone health matter because osteosarcopenia is common and steroid exposure affects both bone and muscle [64,69]. Resistance training is the intervention with the most consistent evidence in sarcopenia, improving muscle mass, strength and physical performance across systematic reviews, and is mechanistically compelling because it activates anabolic signaling [81]; adherence, however, may be limited by fatigue, pain, flares and psychological barriers, which are the same barriers patients with IBD report as limiting physical activity in general [66,67]. Supervised, progressive and symptom-adapted programs are therefore more plausible than generic advice to exercise.

Anti-inflammatory treatment may indirectly improve muscle health by reducing cytokine-driven catabolism and restoring appetite and activity. The emerging question is whether muscle health assessment can be integrated into treat-to-target care. A practical model would screen high-risk patients, interpret existing CT/MRI scans opportunistically, confirm low strength or impaired performance when feasible and refer to dietetics, physiotherapy or prehabilitation. In surgical patients, this approach could be embedded into enhanced recovery pathways; in biologic-treated patients, it could contribute to risk stratification and monitoring.

### 4.4. Knowledge Gaps and Future Directions

The most urgent research need is not another prevalence estimate in isolation but a standardized, outcome-linked diagnostic pathway. A future IBD sarcopenia study should report disease activity, disease duration, medication exposure, steroid use, nutritional status, inflammation, imaging protocol, muscle quantity, muscle quality, handgrip strength and patient-reported function. It should also specify whether sarcopenia was measured during flare, remission, hospitalization, preoperative work-up or biologic initiation. Without this context, prevalence estimates will remain difficult to compare.

Two priorities dominate the resulting research agenda: a standardized, outcome-linked diagnosis and interventional evidence. Table 4 is therefore organized into these two sections, with each remaining gap grouped under the priority it serves. Interventional evidence is the second and, at present, the emptier of the two (Table 4).

The field needs trials that test whether diagnosing and treating sarcopenia changes outcomes. Possible endpoints include strength, SMI, SMD, fatigue, quality of life, flare frequency, hospitalization, postoperative complications, length of stay and treatment persistence. Multimodal programs are likely required because isolated protein supplementation cannot reverse inflammatory catabolism if disease activity remains uncontrolled, and exercise alone may fail if anemia, malnutrition or steroid exposure persist.

### 4.5. Proposed Clinical Pathway

The pathway proposed below deliberately follows the sequential logic of EWGSOP2 and AWGS 2019—trigger, strength, confirmation and severity—rather than proposing a parallel, IBD-specific algorithm. It should be read as the authors’ proposed interpretation of how those consensus frameworks can be operationalized in IBD care and explicitly not as an established IBD-specific recommendation: it has not been prospectively validated, and it is not endorsed by any professional society. A pragmatic pathway for IBD clinics could begin with risk triggers: active disease, low BMI or unintentional weight loss, repeated steroid exposure, fatigue, reduced activity, older age, hospitalization, preoperative assessment, planned biologic initiation, prior surgery or available abdominal CT/MRI. When triggers are present, clinicians could perform handgrip dynamometry and a brief function screen—the strength step of the consensus algorithms—review recent CT/MRI scans for muscle area and quality as the confirmation step and use gait speed or the chair-stand test to grade severity, referring abnormal cases to dietetics and physiotherapy. Where cross-sectional imaging is not clinically indicated, bedside muscle ultrasound performed alongside intestinal ultrasound is a plausible substitute for the confirmation step, subject to the validation caveats set out in Section 3.4.1. Patients awaiting surgery could enter prehabilitation. Patients starting biologics could have muscle status documented as part of baseline risk stratification. Follow-up could repeat strength and weight measures and opportunistically reassess imaging when clinically indicated.

This pathway avoids two extremes: ordering new CT scans solely for sarcopenia and ignoring existing imaging data. It also respects the modern definition of sarcopenia by not equating low SMI with the whole syndrome. In practice, CT or MRI can flag low muscle quantity or myosteatosis, while handgrip and performance testing determine whether the finding has functional expression. The pathway is feasible because it uses tools already present in many IBD settings, but feasibility is not validation, and the evaluation route outlined in Section 4.1 should be completed before it is adopted as practice.

### 4.6. Limitations

This review has limitations. It is a narrative review supported by a structured search, not a registered systematic review. The search was re-run and extended during peer review, which increased the number of eligible diagnostic and prospective studies and corrected the under-retrieval of muscle ultrasound and automated-analysis literature; nevertheless, PubMed/MEDLINE remained the primary database, supplemented by citation tracking, and relevant studies indexed only in other databases may still have been missed. The PRISMA-like counts refer to the screened evidence map for this manuscript and should not be interpreted as an exhaustive systematic-review dataset. Only English-language human studies were included, which may introduce language bias; one included ultrasound study was assessed from its English abstract and structured data because the full text is published in another language. The review is restricted to adults, so none of the prevalence figures, thresholds or prognostic estimates reported here can be applied to children or adolescents. Risk of bias was considered qualitatively rather than scored formally for every empirical study, and no formal quality-appraisal instrument was applied. The IBD-specific ultrasound evidence rests on three single-center prospective studies, so the corresponding section describes potential rather than established practice. The drivers–detection–prognosis–intervention framework and the clinical pathway are expert constructs derived from the reviewed literature; they have not been prospectively validated and should not be read as clinical recommendations. Finally, most included evidence is observational, definitions are heterogeneous, and the associations described should not be interpreted as proven causal effects.

## 5. Conclusions

Sarcopenia in IBD is a frequent, clinically relevant and potentially modifiable systemic complication of the disease, but it remains under-screened and inconsistently defined. Much of the literature describes low muscle mass rather than consensus-defined sarcopenia, and muscle quality—myosteatosis—is a further dimension that carries partly independent prognostic information and is still rarely reported. The literature supports a strong association, though not a demonstrated causal effect, with adverse outcomes, particularly hospitalization, abscess, treatment failure or escalation, surgery-related risk and impaired function. The next step is implementation: standardized IBD-specific thresholds anchored to outcomes, combined imaging and functional assessment applied in the sequential order set out by EWGSOP2 and AWGS 2019, prospective validation of bedside muscle ultrasound as a repeatable and radiation-free monitoring tool, automated opportunistic CT/MRI workflows, and intervention trials integrating inflammation control, nutrition and resistance exercise. The drivers–detection–prognosis–intervention model provides a practical framework—an expert proposal awaiting prospective, multicenter validation—for moving sarcopenia from an overlooked body-composition finding to a measurable target in IBD care.

## Figures and Tables

**Table 1:** Search and selection log for the structured narrative evidence map, after the search was re-run and extended in July 2026.

Selection Step	n	Notes
Records identified and logged	152	PubMed/MEDLINE main strategy (*n* = 118) plus the re-run and extended search of July 2026 targeting ultrasound, diagnostic accuracy, reproducibility and automated analysis (*n* = 26), plus backward/forward citation tracking (*n* = 8)
Duplicates or overlapping records removed	19	Duplicate bibliographic records, records retrieved by both strategies and clear cohort/report overlap
Records screened by title and abstract	133	Broad screening for IBD plus sarcopenia, muscle quality or body-composition relevance
Records excluded at title/abstract stage	67	Non-IBD, unrelated body-composition topic, non-human primary evidence, pediatric-only outside scope or not clinically relevant
Full-text reports assessed	66	Full text or full article page available for eligibility assessment
Full-text reports excluded	12	Non-IBD/no extractable data (*n* = 3); no sarcopenia or muscle outcome (*n* = 2); abstract/editorial only (*n* = 3); overlapping dataset (*n* = 2); pediatric/method-only outside central scope (*n* = 1); insufficient methodological detail (*n* = 1)
Sources included in narrative synthesis	54	39 empirical IBD studies; 5 IBD-specific systematic reviews/meta-analyses; 6 consensus or standardization documents; 4 mechanistic/narrative reviews. The 39 empirical studies and the 5 IBD-specific syntheses (44 sources) are tabulated individually in Table 2; the remaining 10 describe no cohort and are cited in the text. A further 27 background citations identified outside the structured search are not counted here, giving 81 numbered references in total.

**Table 2:** Evidence map of all 44 empirical IBD studies and IBD-specific systematic reviews or meta-analyses included in the narrative synthesis, organized by assessment modality and study objective rather than chronologically. The number of patients analyzed is given for every source; all tabulated studies were conducted in adults, since pediatric-only studies were excluded by the eligibility criteria (Section 2.3). The remaining 10 included sources are consensus, standardization and mechanistic documents that describe no cohort and are cited in the text. For every source the muscle dimension actually measured is stated explicitly, together with the diagnostic definition and cut-off applied, the clinical setting, the key result with adjusted estimates where these were reported and the principal methodological limitation. LMM, low muscle mass (muscle quantity only); MQ, muscle quality (myosteatosis); CDS, consensus-defined sarcopenia (low strength confirmed by low muscle quantity or quality).

Source	Design, Population and Clinical Setting	Muscle Dimension Measured; Definition and Cut-Off	Key Result (Adjusted Estimates Where Reported)	Main Limitation and Interpretation for This Review
**A. Muscle quantity on CT or MRI (low muscle mass) in hospital, surgical and therapy cohorts**				
Adams et al., 2017 [24]	90 adults with IBD starting anti-TNF therapy; USA, single tertiary referral center; retrospective	LMM; CT-derived skeletal muscle index at L3; sex-specific thresholds borrowed from oncology reference populations	Approximately 45% had low muscle mass; in patients with overweight, low muscle mass was associated with subsequent surgery	Retrospective, single center, no strength or performance testing and borrowed cut-offs; highlighted sarcopenic obesity and the failure of BMI-based nutritional assessment
Bamba et al., 2017 [25]	72 adults hospitalized with active IBD (43 CD and 29 UC); Japan, single center; retrospective	LMM; CT-based skeletal muscle index at L3	Low muscle mass was associated with subsequent intestinal resection in admitted patients with CD	Retrospective inpatient sample is enriched for severe disease;; no functional criteria; connects muscle loss to severe disease behavior
Pedersen et al., 2017 [26]	178 adults undergoing IBD surgery; USA, single center, institutional NSQIP dataset; preoperative assessment	LMM; CT-based muscle area at L3	Low muscle mass was associated with surgical morbidity	Retrospective, limited adjustment for albumin, steroids and disease activity; one of the earliest surgical-outcome signals in IBD
Grillot et al., 2020 [27]	88 hospitalized adults with CD; France, single center; abdominal CT	LMM plus visceral adiposity; CT skeletal muscle index and visceral fat area at L3	Low muscle mass and visceral obesity were both associated with adverse outcomes	Retrospective; thresholds not IBD-derived; demonstrates the need to assess muscle and adiposity together rather than separately
Lee et al., 2020 [28]	79 adults with newly diagnosed CD (mean age 29.9 years); South Korea, single center; prevalence and prognosis	LMM; CT-based skeletal muscle index with Asian reference thresholds	Low muscle mass was associated with poorer prognosis in CD	Ethnicity-specific thresholds limit direct transfer to European cohorts; adds East Asian evidence and reinforces population heterogeneity
Boparai et al., 2021 [29]	44 adults with CD; India, single tertiary center; cross-sectional imaging	LMM plus visceral fat; combined CT-derived muscle and visceral fat indices	The combination of low muscle mass and high visceral fat was associated with poor outcomes	Retrospective; combined phenotype defined post hoc; supports sarcopenic obesity as a distinct high-risk phenotype
Ge et al., 2022 [30]	254 adults with acute severe UC and admission CT; China, single center; colectomy decision setting	LMM; CT-based skeletal muscle index at L3	Low muscle mass was evaluated as a predictor of colectomy in acute severe UC	Small, acute, single-center population; fluid shifts may affect measurement; extends relevance beyond CD to severe UC
Hong et al., 2022 [31]	76 adults with CD undergoing surgery; Australia, single center; preoperative CT	LMM; direct comparison of skeletal muscle index and psoas muscle index at L3	Prevalence of low muscle mass was high; SMI and PMI were correlated, but outcomes were not significantly associated with either index	Small sample, wide confidence intervals, no functional assessment; illustrates that prognostic findings are measurement-dependent and not uniformly positive
Nardone et al., 2022 [32]	63 adults with active CD (mean age 44 years) undergoing multidetector CT enterography; Italy, single center	LMM; CT enterography-derived skeletal muscle index at L3	CT-based low muscle mass was associated with clinical outcomes in active CD	Retrospective; active-disease cohort inflates prevalence; uses imaging already routinely acquired in IBD assessment, supporting opportunistic analysis
Nam et al., 2023 [33]	1027 adults with IBD (854 CD and 173 UC); South Korea, tertiary referral cohort; longitudinal clinical course	LMM; CT-based skeletal muscle index	Low muscle mass was associated with a worse IBD clinical course	Registry-based, incomplete nutritional and steroid data; adds regional evidence and supports outcome relevance
Zhang T et al., 2017 [34]	114 adults with CD undergoing bowel resection; China, single center; preoperative CT	LMM; CT-derived skeletal muscle index at L3	Sarcopenia in 70/114 (61.4%); associated with major postoperative complications (OR 9.24, *p* = 0.04)	Retrospective, single center, no confidence interval reported and borrowed cut-offs; an early and much-cited surgical prevalence estimate
Zhang C et al., 2021 [35]	124 adults with CD undergoing intestinal surgery; China, single center; preoperative CT	LMM; CT-derived skeletal muscle index at L3, sex-specific thresholds	Sarcopenia in 34/124 (27.4%); independent risk factor for major postoperative complications (OR 3.97, 95% CI 1.17–13.49, *p* = 0.027)	Retrospective; read together with the preceding row, it shows how far prevalence can move (61.4% versus 27.4%) in two comparable Chinese surgical cohorts using different thresholds
Campbell et al., 2022 [36]	156 adults with IBD (approximately 67% CD; 48% medically treated and 52% surgically); USA, two centers; retrospective	LMM; CT-derived skeletal muscle index at L3	Sarcopenia in 32% of surgical versus 16% of medically treated patients (*p* < 0.02); in the medical cohort, it was associated with subsequent progression to surgery (OR 4.75, 95% CI 1.10–20.57, *p* = 0.04)	Retrospective, modest sample and very wide confidence interval; one of the few datasets comparing medically and surgically managed patients directly
Minawala et al., 2025 [37]	120 adults aged 60 years or older with IBD (60% CD and 40% UC; median age 70 years) undergoing surgery; USA, single institution	LMM; CT-derived skeletal muscle index and total psoas index at L3	Higher skeletal muscle index was associated with fewer adverse 30-day postoperative outcomes (adjusted OR 0.88, 95% CI 0.82–0.94); skeletal muscle index discriminated better than the psoas index (AUC 0.66 versus 0.58, *p* = 0.02)	Retrospective and restricted to older adults, so not generalizable to the young IBD population; useful evidence that whole-slice indices outperform psoas-only measurement
Lee JY et al., 2022 [38]	71 adults with CD (mean age 29.8 years); South Korea, single center; serial CT during treatment	LMM; change in skeletal muscle index at L3 over time	Skeletal muscle index at last follow-up was the only significant predictor of remission (OR 1.21, 95% CI 1.03–1.42, *p* = 0.021)	Retrospective, small, irregular imaging intervals; one of very few longitudinal body-composition datasets in IBD
Celentano et al., 2021 [39]	31 adults with CD undergoing elective ileocecal resection; preoperative MR enterography	LMM on MRI; total psoas area and skeletal muscle area, quartile-based cut-offs	30-day complications in 10/31 (32.3%), rising to 5/8 (62.5%) in the lowest psoas-area quartile, of whom 3 were Clavien–Dindo grade 3 or higher	Very small, quartile-derived cut-offs, no adjusted estimates; shows that MR enterography can be read opportunistically, but the study is underpowered for outcome inference
Kang et al., 2020 [40]	443 adults with IBD and abdominal CT; South Korea, single center; retrospective	LMM; CT-derived skeletal muscle index at L3	Sarcopenia in 34.9%; independently associated with non-alcoholic fatty liver disease (adjusted OR 2.26 after adjustment for age, sex and metabolic syndrome)	Retrospective, no confidence intervals reported, and the outcome is metabolic rather than IBD-specific; links muscle loss to metabolic comorbidity
**B. Muscle quality (myosteatosis) on CT or MRI**				
Cravo et al., 2017 [41]	71 adults with CD and abdominal CT within one month of clinical, laboratory and endoscopic assessment; Portugal, retrospective exploratory study	MQ and LMM; mean muscle attenuation, skeletal muscle index and visceral fat index; sarcopenia by Martin criteria	Higher muscle attenuation was protective against a complicated (stricturing/penetrating) phenotype on multivariable analysis (OR 0.81, *p* = 0.002); high visceral fat index increased risk (OR 26.1, *p* = 0.02); AUC 0.91 for predicting complicated disease	Small, exploratory cross-sectional sample; no confidence intervals reported for the odds ratios; temporality cannot be established; first clear signal that muscle quality adds information beyond muscle area in CD
Pozios et al., 2022 [42]	223 adults undergoing ileocecal resection for CD with adequate preoperative MRI; Germany, single center; median follow-up 48.8 months	MQ and LMM on MRI; myopenia by lowest SMI quartile (20.9 cm^2^/m^2^); myosteatosis by highest quartile of muscle-to-cerebrospinal fluid signal-intensity ratio (0.148)	Neither myopenia nor myosteatosis was associated with anastomotic leak (*p* = 0.363 and *p* = 0.821); SMI was significant univariably for recurrence but not on multivariable analysis (OR 0.951, 95% CI 0.840–1.078, *p* = 0.434)	Retrospective, missing steroid dose and preoperative albumin, quartile-derived cut-offs; the principal negative study; a necessary counterweight to the positive CT literature
Cankurtaran et al., 2023 [43]	116 adults with CD undergoing MR enterography; Turkey, single center; retrospective observational	LMM and MQ on MRI; sarcopenia by SMI < 38.5 cm^2^/m^2^ (women) and <52.4 cm^2^/m^2^ (men); myosteatosis by psoas-to-cerebrospinal fluid signal-intensity ratio > 0.107	Abscess and need for surgery were more frequent with low muscle mass; anti-TNF initiation was more frequent with myosteatosis (*p* = 0.029); need for surgery OR 5.34 (95% CI 1.02–28.03, *p* = 0.047)	Very wide confidence interval, single center, non-standardized MRI myosteatosis threshold; supports MRI as a radiation-free route to muscle quality but not yet as a risk criterion
Donnelly et al., 2024 [44]	124 consecutive adults undergoing resection for CD (2000–2018) with preoperative CT; Ireland, single center	MQ; intermuscular adipose tissue and muscle attenuation at L3; visceral obesity defined as visceral fat area > 163.8 cm^2^ (men)/>80.1 cm^2^ (women)	Intermuscular adipose tissue was independently associated with postoperative morbidity (OR 1.08, 95% CI 1.01–1.16, *p* = 0.037) and a higher comprehensive complications index (*p* = 0.029); adiposity alone did not increase overall morbidity	Retrospective, single center, 18-year accrual spanning major changes in biologic therapy; the strongest evidence that myosteatosis carries prognostic value independent of obesity
Xiong et al., 2025 [45]	157 adults with CD (42 penetrating and 115 non-penetrating) treated non-surgically for at least one year; China, retrospective CT-enterography cohort	MQ and LMM; automated segmentation deriving a skeletal muscle ratio = muscle/(muscle + intermuscular adipose tissue); sarcopenia by muscle area at L3	Skeletal muscle ratio discriminated treatment escalation better than conventional sarcopenia metrics (AUC 0.82 penetrating, 0.92 non-penetrating; *p* = 0.002 and *p* < 0.001) and was an independent protective factor (combined HR 0.64, 95% CI 0.49–0.82, *p* = 0.001)	Retrospective, no external validation, cut-off derived in-sample, small penetrating subgroup with a very wide hazard ratio; bridges the myosteatosis and automated-analysis literature
**C. Muscle ultrasound**				
Mulinacci et al., 2024 [17]	Prospective two-cohort study; 100 consecutive patients for feasibility and reliability and 53 adults with IBD (34 CD and 19 UC) for diagnostic accuracy; Italy, single center; sarcopenia prevalence 50%	LMM by ultrasound; rectus femoris (thickness and cross-sectional area), rectus abdominis and biceps brachii thickness at defined landmarks; ultrasound muscle index = sum of three thicknesses/height^2^; reference standard BIA, second comparator MRI	Inter- and intra-observer ICC > 0.95 (maximum 0.97–0.98 for rectus femoris cross-sectional area); ultrasound versus MRI for rectus abdominis ICC 0.96; AUC 0.85 (rectus abdominis), 0.80 (biceps brachii and rectus femoris thickness) and 0.81 (ultrasound muscle index) versus SARC-F 0.54 and chair-stand test 0.76	Single center, small accuracy cohort, no outcome linkage, BIA reference thresholds derived from Asian populations and no SARCUS quality parameters; the key IBD validation study and the basis for positioning ultrasound as a screening tool
Akchurina et al., 2025 [19]	102 adults with IBD (49% UC and 51% CD) and 10 controls; hospital-based comparative study, gastroenterology inpatient setting	LMM by ultrasound; mid-upper arm and mid-thigh muscle thickness, compared with anthropometric circumferences, bioimpedance and handgrip dynamometry	Ultrasound-measured mid-thigh thickness was lower in patients with IBD than in controls (women 31.05 versus 41.30 mm, *p* < 0.05) and correlated with circumference, bioimpedance-derived skeletal muscle mass and grip strength	Descriptive rather than diagnostic; no cut-offs, no accuracy statistics and no reliability data reported; small control group; published in Russian with an English abstract; supports construct validity only
Palmisano et al., 2026 [18]	353 adults with IBD screened by SARC-F, of whom the 57 at risk (SARC-F ≥ 4) underwent muscle ultrasound; Italy, single center, outpatient	LMM by ultrasound; psoas muscle thickness-to-height ratio and diaphragm thickness-to-height ratio; literature threshold of 16.8 mm/m cited for psoas	Mean psoas thickness-to-height ratio 16.62 mm/m; diaphragm and psoas indices were correlated (r = 0.36, *p* < 0.05), and the association persisted after adjustment (beta 0.018, 95% CI 0.005–0.030, *p* = 0.008); inter- and intra-rater ICC > 0.95	No diagnostic accuracy reported, threshold borrowed rather than derived, SARC-F-gated design misses patients with low SARC-F and no SARCUS quality parameters; proposes the diaphragm as an accessible surrogate site
**D. Consensus-defined sarcopenia (strength and muscle mass, with or without physical performance)**				
Unal et al., 2021 [46]	344 adults with IBD in clinical remission; Turkey, single center; cross-sectional outpatient study	CDS; malnutrition screening combined with strength and body-composition assessment	Malnutrition and sarcopenia remained prevalent despite clinical remission	Cross-sectional, single center; shows that symptom remission does not exclude muscle disease and justifies screening outside flare
Liu et al., 2022 [47]	110 adults with IBD (85 UC and 25 CD), aged 18–60 years; China, prospective single-center cohort; AWGS 2019-style assessment	CDS; AWGS 2019 criteria combining grip strength, muscle mass and physical performance	Pre-sarcopenia 44.6% and sarcopenia 50.8%; sarcopenia was associated with poor clinical outcomes	AWGS thresholds are Asian-specific, so the high prevalence is not transferable to European cohorts; important prospective evidence using function-oriented criteria
Neelam et al., 2024 [48]	114 adults with UC (mean age 36.5 years); India, prospective single-center cohort; strength, performance and muscle mass measured concurrently	CDS; probable, confirmed and severe sarcopenia defined sequentially	Probable sarcopenia 37.7%, sarcopenia 21.9% and severe sarcopenia 12.2%; associated with disease activity and lower BMI	Single center, no long-term outcome follow-up; demonstrates substantial burden in UC and the added value of applying the full sequential algorithm
Dharap et al., 2026 [49]	117 adults with IBD (73 UC, 42 CD and 2 IBD-unclassified); India, prospective follow-up cohort; outpatient with structured follow-up	CDS; strength plus muscle mass, with severity grading	Sarcopenia 40.2% and severe sarcopenia 8.5%; freedom from flare was markedly lower with sarcopenia (5.3% versus 46.1%)	Single center, modest sample and limited adjustment for baseline disease activity; links baseline sarcopenia to short-term flare risk, an outcome rarely reported
Dermine et al., 2025 [50]	60 adults with IBD (52% CD and 48% UC; median age 37 years), most in remission; France, prospective outpatient cohort	CDS and LMM; EWGSOP2-style criteria distinguishing sarcopenia, probable sarcopenia and myopenia	Sarcopenia 10%, probable sarcopenia 18% and myopenia 20%	Small sample and a stable population, so estimates are not generalizable to inpatients; the clearest demonstration that prevalence falls when modern functional criteria are applied to stable outpatients
**E. Body composition and response to biologic therapy**				
Ding et al., 2017 [51]	106 anti-TNF-naive adults with CD; UK, single tertiary center; body composition measured before starting therapy	LMM; CT-derived body-composition variables including lean mass	Body-composition variables were associated with primary non-response and loss of response to anti-TNF therapy	Observational; drug exposure and albumin not fully accounted for; suggests a pharmacokinetic as well as prognostic role for lean mass
Holt et al., 2017 [52]	68 adults with IBD at anti-TNF initiation; Australia, retrospective single-center analysis	LMM; CT-derived low muscle mass at treatment initiation	Low muscle mass was associated with early anti-TNF treatment failure	Retrospective, definitions varied, no functional testing; supports muscle assessment before biologic initiation as risk stratification, not as a dosing criterion
Grova et al., 2023 [53]	358 adults with CD starting biologics between 2014 and 2020 with CT or MRI within 6 months; Italy, two centers	LMM; psoas muscle index <5.4 cm^2^/m^2^ in men and <3.56 cm^2^/m^2^ in women	Sarcopenia in 18.2%; endoscopic remission at 12 months in 14.8% versus 47.7% (*p* = 0.002); independent predictor of failure to achieve endoscopic remission (OR 5.2, *p* = 0.006); no association with steroid-free clinical remission, hospitalization or surgery	Retrospective; psoas-only index; the published abstract reports no confidence interval for the odds ratio; the only study linking muscle status to an endoscopic endpoint
Liu J et al., 2023 [54]	94 adults with CD receiving biologic therapy; China, single center; CT or MRI	LMM; skeletal muscle index at L3 on CT or MRI	Loss of response in 57.4%; sarcopenia associated with loss of response (OR 3.89, 95% CI 1.31–11.54), and with loss of response to infliximab specifically (OR 3.31, 95% CI 1.11–9.87)	Retrospective, modest sample, CT and MRI definitions pooled; supports the pharmacokinetic-marker hypothesis without testing it directly
Fang Y et al., 2024 [55]	269 adults with moderate-to-severe CD treated with infliximab or ustekinumab, plus 172 appendicitis controls; China, single center; propensity-score matched	LMM; CT-derived low muscle mass at baseline	Low muscle mass was associated with lower clinical response and remission at weeks 8–14 and with lower remission at weeks 24–30 and 52 and was independently associated with loss of response at weeks 24–30 and 52; no effect estimate is reported for these outcomes in the published abstract	Retrospective; the analytic sample after propensity-score matching is not stated, and the headline efficacy findings are reported narratively without effect estimates
**F. Systematic reviews and meta-analyses**				
Ryan et al., 2019 [8]	Systematic review; 5 studies, 658 adults with IBD (approximately 70% CD)	Predominantly LMM; pooled across heterogeneous CT-based definitions	42% pooled prevalence of myopenia (sarcopenia proper 17%); three studies reported a higher probability of surgery and more frequent major postoperative complications	Very small evidence base at the time; definitions pooled despite heterogeneity; established that low muscle mass is common and clinically meaningful in IBD
Potcovaru et al., 2023 [13]	Systematic review; 16 studies in adults with IBD; pooled patient total not reported (component studies ranged from 19 to 11,001 patients)	Mixed LMM and CDS; the review’s explicit purpose was to compare diagnostic criteria and cut-offs across studies	Diagnostic criteria, imaging levels and cut-offs were too heterogeneous to pool, and no summary prevalence or effect estimate could be derived	No meta-analysis was possible; the clearest published demonstration that IBD sarcopenia definitions are not comparable across studies
Fatani et al., 2023 [14]	Systematic review; 35 studies in adults with IBD (34 contributing prevalence data, 20 outcome data, 17 nutritional data); pooled patient total not reported	Mixed LMM and CDS, reported separately as myopenia, pre-sarcopenia and sarcopenia	Myopenia 42%, pre-sarcopenia 34% and sarcopenia 17%; myopenia was associated with therapy failure, postoperative complications and lower bone mineral density	Pooled across heterogeneous definitions and settings; the source of the widely quoted 42%/17% split between myopenia and sarcopenia proper
Feng et al., 2024 [15]	Systematic review and meta-analysis; 17 studies, 2895 adults with IBD	Mixed LMM and CDS; definitions not harmonized across included studies	Sarcopenia associated with treatment failure (OR 2.00, 95% CI 1.43–2.79) and need for surgery (OR 1.54, 95% CI 1.06–2.23); subgroup effects for corticosteroids and individual biologics were inconsistent	Pooling of heterogeneous definitions; observational studies with variable adjustment; the most direct synthesis available for treatment failure
Saleh et al., 2025 [16]	Systematic review and meta-analysis restricted to CD; 14 studies, 2334 adults	Predominantly LMM on CT	Higher hospitalization risk (OR 1.87, 95% CI 1.19–2.93) and abscess risk (OR 5.03, 95% CI 2.05–12.38); no significant pooled effect on surgery, loss of biological response, need for biologics or surgical-site leak	Residual confounding by disease severity; wide interval for abscess; shows a robust but clearly outcome-specific prognostic signal
**G. Biomarkers and automated or artificial intelligence-assisted analysis**				
Godala et al., 2024 [56]	82 adults with IBD (48 CD and 34 UC; mean age 38.1 years) and 25 healthy controls; Poland, single center; case control	Molecular phenotype; serum myostatin and activin A alongside muscle mass index and grip strength	Lower myostatin and activin A patterns were reported in IBD with sarcopenia; myostatin correlated with muscle mass index and handgrip strength	Small sample, cross-sectional, no outcome data, assay variability; biomarkers remain promising but are not ready for routine diagnosis
Fang et al., 2026 [57]	308 hospitalized adults with IBD (251 UC and 57 CD; training 217, validation 91); China, single center; CT-derived body composition with machine learning modeling	LMM and MQ; skeletal muscle index and skeletal muscle density at L3	Sarcopenia and myosteatosis were independently associated with treatment escalation; a LightGBM model achieved a validation AUC of 0.763	Retrospective, internal validation only, inpatient population; illustrates the shift towards automated imaging analysis and risk prediction
Chen et al., 2025 [58]	134 adults with CD treated with infliximab (training 84 and validation 50); China, two institutions; retrospective	Radiomic phenotype of the psoas muscle on CT; 20 differential radiomic features across seven machine learning algorithms	Mean validation AUC 0.849 across models; best-performing extreme gradient boosting model AUC 0.910	Small sample relative to the number of features and algorithms; internal split rather than true external validation; radiomic features are scanner- and protocol-sensitive; psoas-only region of interest
Gupta et al., 2026 [21]	Deep learning pipeline trained on 550 CT scans (6516 slices) and tested on 601 scans from adults with acute pancreatitis, IBD, gallbladder cancer or biliary obstruction; India, single center	LMM; automated L3 localization followed by nnU-Net skeletal muscle segmentation, with sarcopenia defined by conventional SMI thresholds	Dice 0.93–0.97; expert-rated excellent muscle segmentation in 90–93%; sarcopenia detection sensitivity 0.94–0.97, specificity 0.84–0.97 and AUC up to 0.92	Retrospective, no prospective outcome linkage, and the reference standard still relies on borrowed SMI cut-offs, so an accurate model may reproduce an imprecise definition

**Table 3:** Diagnostic modalities for sarcopenia and muscle health assessment in IBD, with the position each modality occupies in the sequential EWGSOP2 and AWGS 2019 diagnostic algorithms.

Modality	Main Dimension and Position in the Sequential EWGSOP2/AWGS 2019 Algorithm	Practical Advantages	Limitations in IBD
CT at L3/SMI	Muscle quantity, as well as muscle attenuation for quality; confirmation step only—never sufficient alone for a diagnosis of sarcopenia	Often already available in IBD; objective; supports retrospective prognosis	Radiation if newly acquired; cut-offs borrowed; affected by software, contrast phase and BMI; attenuation thresholds for myosteatosis are inconsistent across studies; not a strength test
Psoas muscle index	Simplified muscle quantity proxy; confirmation step, with lower validity than whole-slice analysis	Fast and easy; feasible in busy radiology workflows	Less representative than whole-slice muscle; inconsistent thresholds
MRI/MR enterography	Muscle quantity and, in some protocols, quality; confirmation step	Radiation-free; common in CD monitoring	Less standardized; segmentation time; availability/cost
DXA	Appendicular lean mass and bone density; confirmation step and the reference method in most non-IBD sarcopenia research	Useful when bone disease is also assessed; low radiation	Limited muscle quality data; not routine for acute IBD decisions
BIA	Estimated fat-free mass/skeletal muscle mass; confirmation step where imaging is unavailable and the confirmation method used in community settings by AWGS 2019	Cheap, rapid, clinic friendly	Sensitive to hydration and active inflammation; equation dependent
Ultrasound	Regional muscle thickness and cross-sectional area (quantity) and echo intensity (quality); a screening tool in IBD at present and a potential confirmation tool once IBD-specific thresholds exist	Portable, no radiation, potential bedside tool	Operator dependent; no IBD-specific cut-offs; validated in IBD by three single-center prospective studies only; quality parameters (echo intensity and pennation angle) untested in IBD; possible influence of edema in active disease
Automated/AI-assisted CT or MRI segmentation	Muscle quantity and quality extracted without manual input; an enabling technology for the confirmation step rather than a separate diagnostic criterion	Removes the segmentation bottleneck; Dice > 0.93 reported in gastrointestinal cohorts; makes opportunistic reporting of every abdominal scan feasible	Mostly retrospective and internally validated; radiomic features are scanner-dependent; trained against borrowed cut-offs; not yet integrated into radiology reporting workflows
Calf circumference	Surrogate for muscle quantity; the case-finding step in the AWGS 2019 community pathway and the added component of SARC-CalF	Requires only a tape measure; validated as a screening trigger in community settings	Affected by edema and adiposity, both common in IBD; no IBD-specific thresholds
Handgrip dynamometry	Muscle strength; the assessment step and the entry point of both algorithms—low grip strength alone establishes probable (EWGSOP2) or possible (AWGS 2019) sarcopenia	Central to modern definitions; fast and inexpensive	Needs protocol and reference values; may miss lower-limb dysfunction
Gait speed, chair stand, SPPB and TUG	Physical performance; severity grading, except for the chair-stand test, which may also serve as a strength measure at the assessment step	Captures functional severity and frailty overlap	May be normal in young patients despite low muscle mass
SARC-F/SARC-CalF	Screening for functional impairment; the case-finding step that triggers the algorithm	Very practical for clinics	Low sensitivity in early or non-geriatric sarcopenia
Biomarkers: myostatin, activin A, IGF-1, irisin and inflammatory markers	Potential molecular phenotype; no position in the current algorithms	May support early risk stratification	Not validated as standalone diagnostic criteria in IBD

**Table 4:** Literature gaps and research priorities, grouped under the two priorities identified in the text: standardized, outcome-linked diagnosis, and interventional evidence.

Gap	Why It Matters	Recommended Next Step
**Priority 1. Standardized, outcome-linked diagnosis**		
IBD-specific diagnostic thresholds	Most thresholds are borrowed from geriatric, oncologic or general-population cohorts.	Prospective cohorts should derive sex-, age-, ethnicity-, BMI- and disease context-specific thresholds linked to outcomes.
Muscle quality and myosteatosis	SMI alone may miss adverse muscle composition, myosteatosis carries partly independent prognostic information, and no consensus numeric definition currently exists, with CT attenuation thresholds and MRI signal-intensity ratios not being equivalent measurements.	Report muscle attenuation or skeletal muscle density and intermuscular adipose tissue alongside muscle area, state the Hounsfield unit window used, and work towards a consensus threshold that is comparable across CT, MRI and ultrasound echo intensity.
Functional validation	Many imaging studies do not measure handgrip strength or performance.	Combine imaging with strength and patient-reported function.
Muscle ultrasound standardization and validation in IBD	Ultrasound is the only modality that is simultaneously low-cost, portable, radiation-free and repeatable, yet IBD validation rests on three single-center studies, no IBD-specific cut-offs exist, and no study has linked ultrasound-defined muscle status to clinical outcomes.	Multicenter prospective studies applying SARCUS acquisition standards, reporting inter- and intra-observer reliability and diagnostic accuracy against consensus criteria, deriving IBD-specific thresholds, adding quality parameters such as echo intensity and linking findings to surgery, hospitalization, treatment failure and flare-free survival.
Nutritional and dietary assessment	Malnutrition, reduced intake and micronutrient deficiency are central to the pathogenesis described in this review, yet few IBD sarcopenia studies report validated nutritional assessment, quantified dietary intake or specific deficiencies, so the nutritional contribution to muscle loss cannot be separated from that of inflammation.	Report GLIM-defined malnutrition, quantified protein and energy intake and vitamin D and other micronutrient status alongside every muscle measurement; test formally whether nutritional adequacy modifies the association between muscle status and outcome.
Sarcopenic obesity	BMI can hide muscle loss, and visceral adiposity may interact with inflammation.	Study combined muscle–fat phenotypes and biologic pharmacokinetics.
Automation and artificial intelligence	Manual segmentation limits routine use, and the automated models published so far are largely retrospective and internally validated, trained against borrowed cut-offs.	Validate automated CT/MRI segmentation and risk models in external, prospective IBD cohorts, and evaluate automated body-composition reporting as an implementation strategy—for example, an automatically generated field in every abdominal CT or MR enterography report—with management change as the endpoint.
Under-represented regions	Many cohorts come from limited geographic settings.	Include Eastern Europe, Romania and other under-represented regions to improve external validity.
**Priority 2. Interventional evidence and implementation**		
Longitudinal causality	Observational associations cannot distinguish cause from severity marker.	Repeated body-composition, inflammatory and outcome measurements before and after treatment.
Intervention trials	No large IBD-specific trials test sarcopenia reversal as an endpoint.	Randomized or pragmatic trials of dietetic care, resistance training, prehabilitation and multimodal programs.
Implementation and validation of the proposed framework and pathway	The drivers–detection–prognosis–intervention framework and the clinical pathway proposed here are expert constructs; neither has been prospectively validated, and it is unknown whether applying them changes patient outcomes or is deliverable within routine consultation time.	A three-stage evaluation: a prospective multicenter cohort measuring all four detection domains concurrently to derive outcome-anchored thresholds; longitudinal follow-up to test and externally validate a framework-derived risk score; and a stepped-wedge or cluster-randomized implementation trial with clinical and patient-reported primary endpoints and explicit feasibility measures.

## Data Availability

No new data were generated or analyzed in this review. The evidence map is available within the article.

## Abbreviations

AI, artificial intelligence; AUC, area under the curve; AWGS, Asian Working Group for Sarcopenia; BIA, bioelectrical impedance analysis; BMI, body mass index; CD, Crohn’s disease; CI, confidence interval; CSA, cross-sectional area; CT, computed tomography; DXA, dual-energy X-ray absorptiometry; EWGSOP, European Working Group on Sarcopenia in Older People; GLIS, Global Leadership Initiative in Sarcopenia; HR, hazard ratio; HU, Hounsfield units; IBD, inflammatory bowel disease; ICC, intraclass correlation coefficient; IMAT, intermuscular adipose tissue; MRI, magnetic resonance imaging; OR, odds ratio; PMI, psoas muscle index; SARC-F, Strength, Assistance in walking, Rise from a chair, Climb stairs and Falls questionnaire; SARC-CalF, SARC-F plus calf circumference; SARCUS, standardized muscle ultrasound working group; SMD, skeletal muscle density; SMI, skeletal muscle index; SPPB, Short Physical Performance Battery; TUG, Timed-Up-and-Go; UC, ulcerative colitis; US, ultrasound.
